# Polycyclic Aromatic Hydrocarbons’ Impact on Crops and Occurrence, Sources, and Detection Methods in Food: A Review

**DOI:** 10.3390/foods13131977

**Published:** 2024-06-22

**Authors:** Tengfei Liu, Li Zhang, Leiqing Pan, Daifeng Yang

**Affiliations:** 1 College of Food Science and Technology, Nanjing Agricultural University, Nanjing 210095, China; liutengfei@jaas.ac.cn; 2 Jiangsu Taihu Area Institute of Agricultural Sciences, Suzhou 215106, China; 3 Suzhou Vocational University Center for Food Safety and Nutrition, Suzhou 215104, China; zhangli_szd@163.com

**Keywords:** polycyclic aromatic hydrocarbons, crops, food, sources, contamination, detection

## Abstract

Polycyclic aromatic hydrocarbons (PAHs) represent a category of persistent organic pollutants that pose a global concern in the realm of food safety due to their recognized carcinogenic properties in humans. Food can be contaminated with PAHs that are present in water, air, or soil, or during food processing and cooking. The wide and varied sources of PAHs contribute to their persistent contamination of food, leading to their accumulation within these products. As a result, monitoring of the levels of PAHs in food is necessary to guarantee the safety of food products as well as the public health. This review paper attempts to give its readers an overview of the impact of PAHs on crops, their occurrence and sources, and the methodologies employed for the sample preparation and detection of PAHs in food. In addition, possible directions for future research are proposed. The objective is to provide references for the monitoring, prevention, and in-depth exploration of PAHs in food.

## 1. Introduction

**Figure 1:**
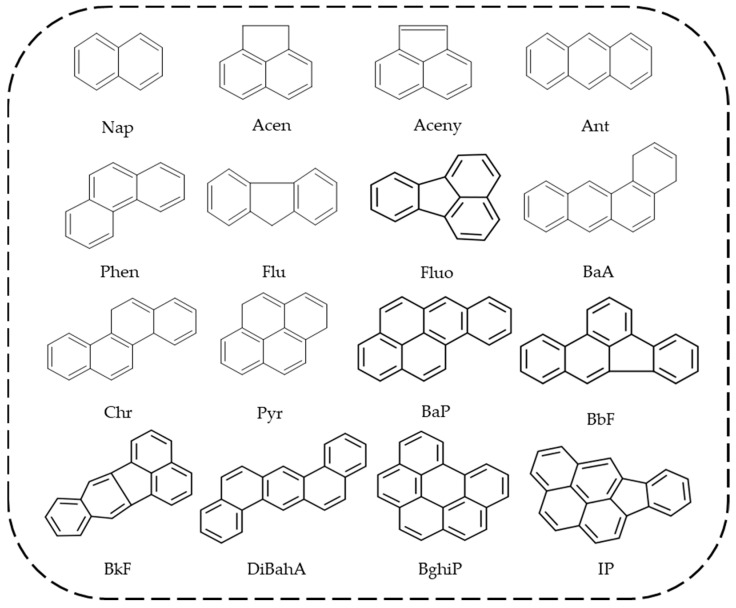
Structures of the 16 EPA PAHs.

Polycyclic aromatic hydrocarbons (PAHs) are hydrophobic compounds that contain two or more fused aromatic rings in their molecular structure. They are prevalent pollutants found in various environmental matrices such as air, water, and soil [1,2,3,4]. To date, over 100 types of PAHs have been identified in the environment at substantial concentrations [5]. Of the numerous PAH compounds, 16 PAHs have been identified as priority pollutants for environmental regulation and control by the US Environmental Protection Agency (EPA). Benzo[a]pyrene (BaP) due to its significant cancer-causing potential has been classified as a Group 1 carcinogen by the International Agency for Research on Cancer (IARC), and is frequently employed as an indicator for PAH contamination [6]. Table 1 presents the principal physical–chemical properties of the 16 priority PAHs, with their structures depicted in Figure 1. These compounds are highly persistent, and recent studies have documented their widespread occurrence in various food products globally [7,8,9,10]. It is widely confirmed that PAHs are toxic to human health, which can cause severe health issues such as carcinogenic, teratogenic, and mutagenic effects [11,12,13,14]. Consequently, the level of PAHs in food is a matter of public concern, given that food consumption represents the major route of PAH exposure for the general populace, particularly non-smokers and non-occupationally exposed individuals [15]. Many nations have implemented regulatory directives to control the concentrations of PAHs in food via the establishment of maximum levels for BaP and/or PAH4 (BaP + BaA + BbF + Chr) in different food products, as detailed in Table 2. Thus, the monitoring of PAH levels in food products is essential for compliance with regulatory standards and for ensuring that people are not exposed to PAH concentrations that are detrimental to their health.

Over the past decade, methods for PAH analyses in food have been extensively developed and evaluated worldwide. Due to the complexity of food-related samples, the quantification of PAHs in such matrices often requires the use of a sample preparation procedure followed by chromatographic separation prior to detection. Gas chromatography and liquid chromatography (GC and LC, respectively) are prominent techniques used to determine PAHs in food [16], while some other technologies such as immunoassays and sensors are also utilized [17,18]. Owing to the low levels and dynamic variations in PAHs in food samples, the methodologies applied for their detection are constantly evolving. There have been excellent review papers focusing on the determination of PAHs in food. For example, Meng et al. [19] provided an overview of pretreatment methods for detecting PAHs in edible oils. Bansal et al. [20] reviewed the quantification techniques for the estimation of PAHs in food products. However, these reviews do not cover the impact of PAHs on crops and their occurrence and sources in food. This review aims to discuss the up-to-date knowledge on the impacts of PAHs on crops, the occurrence and sources of PAHs in food, and sample preparation and analytical techniques for determining PAHs in food, in order to provide valuable references for monitoring, prevention, and in-depth exploration of PAHs in food.

**Table 2:** Maximum levels for BaP and PAH4 in food of different nations.

Nation	Types of Food	BaP (μg/kg)	PAH4 (μg/kg)	Ref.
European Union	Oil and fats intended for direct human consumption or use as an ingredient in food	2.0	10.0	[21]
	Cocoa bean and derived products	5.0	30.0	
	Coconut oil intended for direct human consumption or use as an ingredient in food	2.0	20.0	
	Smoked meat and smoked meat products	2.0	12.0	
	Muscle meat of smoked fish and smoked fishery products	2.0	12.0	
	Smoked sprats and canned smoked sprats; bivalve mollusks (fresh, chilled or frozen)	5.0	30.0	
	Bivalve mollusks (smoked)	6.0	35.0	
	Processed cereal-based food and baby food for infants and young children	1.0	1.0	
	Infant formulae and follow-on formulae, including infant milk and follow-on milk	1.0	1.0	
	Dietary foods for special medical purposes intended specially for infants	1.0	1.0	
China	Cereals/cereal products	2.0	—	[22]
	Meats/meat products	5.0	—	
	Aquatic foods/aquatic food products	5.0	—	
	Milk and dairy products	10.0	—	
	Oils and fats and their products	10.0	—	
Korea	Dried and smoked fish	10.0	—	[23]
Canada	Olive pomace oils	3.0	—	[24]
Brazil	Olive pomace oils	2.0	—	[25]

—, no data.

## 2. Impacts of PAHs on Crops

Crops, during their growth period, are inevitably exposed to and accumulate contaminants such as PAHs from the surrounding environment. Limited research has indicated that PAHs can impact crop growth influencing major physiology including germination, morphology, photosynthesis, and enzyme activity [26,27,28]. Li et al. conducted a hydroponic experiment to explore the impacts of BaP on the growth and antioxidant enzyme activities of wheat (*Triticum aestivum* L.) seedlings. The study revealed that wheat seedlings treated with 54 mg/L of BaP exhibited a dramatical decrease in biomass and plant height compared to the control (CK), with both above-ground fresh mass and below-ground dry mass being suppressed. Moreover, under BaP stress, the superoxide dismutase (SOD) activity presented a trend of increase–decrease–increase and was lower than that in CK, whereas the peroxidase (POD) and catalase (CAT) activities decreased after an initial increase [29]. Similarly, Wang et al. investigated the effects of Fluo and BaP on growth, physiological performance, and quality of rape (*Brassica chinensis*), as well as their patterns of accumulation in stem and leaves. They found that the accumulated concentrations of Fluo and BaP in rapes increased with the levels of Fluo and BaP in the soil. The accumulations of Fluo in stems and leaves varied significantly (*p* < 0.05) among treatments with different rates of Fluo addition. When compared to CK, the accumulation of BaP in rapes significantly increased in treatments with 5.0 and 10.0 mg/kg of BaP, with the highest amount of accumulation at 10 mg/kg still below the national food security standard. Low concentrations of Fluo and BaP had positive effects on leaf length, leaf width and above-ground biomass; while high concentrations had negative effects. As the levels of Fluo and BaP increased, plant height and photosynthetic rate significantly decreased compared to CK. The chlorophyll content initially increased at lower concentrations but declined at higher concentrations. The study also found that the reduced vitamin C was inhibited by Fluo, with the lowest levels observed at 5.0 mg/kg of Fluo. On the other hand, the reduced vitamin C varied irregularly with BaP treatments; it increased slightly at 0.5 mg/kg of BaP and was lowest at 5.0 mg/kg of BaP compared with CK [30]. Further, Cai et al. reported that Pyr had significant effects on root dry weight but little effect on shoot dry weight and plant height of Chinese cabbage. The whole plant dry weight decreased with the reduction in root dry weight. High concentrations of Pyr (>201.3 mg/kg) resulted in retarded growth, delayed seed germination, decreased leaf area, decreased shoot height, chlorosis, and ultimately lower yields [31].

Interestingly, despite being hazardous pollutants, PAHs have been found to potentially enhance the quality and yield of crops within a specific concentration range. When *B. parachinensis* was treated with 600 μg/kg of PAHs through root hydroponics, there was an increase in its cellulose, vitamin C, and soluble protein levels. Similarly, leaf smear treatment with the same dose of PAHs enhanced both the single plant weight and soluble sugar content in *B. parachinensis*, while facilitating the accumulation of organic matter, resulting in higher yields and improved food quality [32]. PAH treatment also affects enzyme activities in *B. parachinensi*. For instance, a low-concentration (73.9 μg/kg) PAH treatment enhances the activities of CAT and POD, whereas a medium-concentration (147.8 μg/kg) treatment increases the SOD activities [33]. These alterations contribute to a reduction in the accumulation of PAHs in *B. parachinensi* and an improvement in its quality [33]. However, the mechanism of action remains poorly understood. Previous studies propose that the structural similarity between PAHs and hormones like phytoglucomannan may be responsible for their growth-promoting actions [34]. The impacts of PAHs on crops are intricate, and limited studies have been conducted to date. Therefore, further and more in-depth studies are required in this area.

## 3. Sources of PAHs in Food

**Figure 2:**
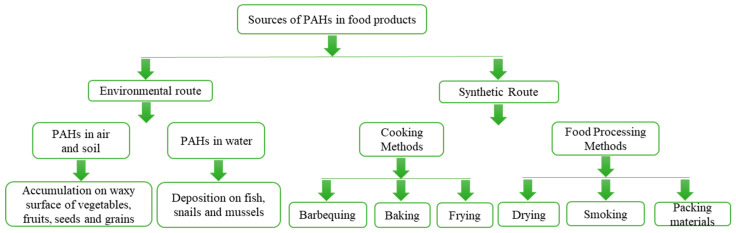
Main sources of PAHs in food products.

As environmental contaminants, PAHs can enter our food system through a variety of pathways [35], as depicted in Figure 2. Due to their recalcitrance to breakdown and long-range migration ability, PAHs persistently contaminate the environments essential for crop cultivation, such as air, water, and soil, and enter food crops through root uptake or via leaf absorption, and subsequently accumulate within the food chain [36]. Leaf absorption has been shown to be the main pathway for plants to assimilate PAHs [37,38,39]. Over 40% of the PAHs released into the atmosphere come into contact with plant leaves through wet and dry deposition [40] and are effectively accumulated in plant leaf cuticles, which are hydrophobic lipid structures [41]. Stomata, tiny pores on the leaf surface responsible for gas exchange, serve as the main entry points for PAHs. They are the most active and prominent components of plant tissues for adsorbing these contaminants. PAHs can either adhere to stomatal openings or be directly absorbed through the stomatal pores, thereby gaining access to the plant’s internal tissues [42]. Typically, PAH concentrations in plant leaves are twice as high or even higher compared to plant roots [39,43]. Also, there is considerable variation in PAH levels among different types of vegetables, with leafy vegetables having the highest concentrations, followed by root and fruit vegetables [44]. This disparity can be attributed to the larger surface area of the leafy parts, which facilitates atmospheric deposition. Aquatic creatures, such as fish, shellfish, and shrimp, are also susceptible to PAH exposure through water and sediment, resulting in bioaccumulation [45,46]. When these plants or animals are used as raw materials in food processing, they can contaminate the food and pose severe health risks to humans.

PAHs also occur in food as a result of certain processing and cooking methods such as frying, grilling, baking, barbecuing, and smoking. During these high-temperature (150–400 °C) thermal processes, proteins, fats, and carbohydrates in the food are pyrolyzed, oxidized, and polymerized to produce PAHs [36]. One study reported that that pan frying caused lower levels of BaP (1.39 versus 1.62 μg/kg) and PAH4 (5.58 versus 5.73 μg/kg) in beef meat than barbecuing [47]. The total concentration of 16 EPA PAHs increased nearly three times during the frying of fish slices, with some PAHs such as Nap, Fluo, and Pyr showing a five-fold increase [48]. A two- to six-fold increment was observed in PAH levels when flour was baked [49]. Similarly, the level of BaP increased from 30.56% in uncooked food to 61.52% in cooked ones [50]. Previous studies revealed that the amounts of PAHs formed in food products are affected by multiple factors [23,51,52,53,54], such as the inherent properties of food materials (e.g., pH, composition, and moisture), cooking duration, and the heat source (e.g., coal, gas, wood, electrical) and temperature used. For instance, among different roasting techniques (charcoal, gas, and electric), charcoal roasting was reported to contribute significantly to PAH formation [55,56,57]. In the case of gas roasting, the PAH4 content in beef surpassed the EU-regulated limits when the temperature exceeded 300 °C [58]. The pH of marinating sauces used in grilling chicken affects PAH formation. A sauce with a pH of 7.51 significantly promotes PAH formation, with the PAH4 content increasing more than four-fold compared to a sauce with a pH of 5.35 [59]. According to a recent study, the moisture content of firewood positively correlated with PAH generation during the production of katsuobushi, a traditional smoked-dried bonito in Japan [23].

Furthermore, the migration of PAHs from food contact materials (e.g., plastic, rubber and paper) can also lead to food contamination [60]. A recent investigation into four different types of food contact materials available in the Chinese market, including laminated film bags, nylon tableware, food containers, and disposable tableware, revealed the presence of PAHs in all of these materials [61]. These PAHs were mainly related to Nap, Phen, Fluo, and Pyr, with Phen exhibiting the highest rate that exceeded the standard rate at 77.1%, possibly due to the use of inferior raw materials during production [61]. In a study of 20 commercially paper-based food contact materials, including fast food packaging cartons, instant noodle bags, food packaging bags, and paper cups, high levels of Nap, Flu, Phen, and Ant were found in a yellow packaging paper bag, with Ant contents reaching up to 201,400 μg/kg [62]. When food contact materials are contaminated with PAHs, these substances can migrate into the food and be ingested by humans, resulting in PAH exposure-related health hazards [63,64].

## 4. Occurrence of PAHs in Food

The widespread contamination of food by PAHs has been occurring for years due to human activities, leading to their frequent detection in food products. A recent study by Einolghozati et al. investigated the pollution of processed cereals with PAHs across Europe, Africa, America, and Asia from 1972 to 2021. Results revealed that PAHs were found in bread, spaghetti, flour, and bran in all of these regions. The dominant PAHs were Ant, Chr, Flu, and Nap, with concentrations ranging from 0.39 to 2.95 μg/kg [65]. Although the measured levels are generally low, the high consumption of processed cereals renders them a considerable source of PAH exposure in humans. In another study, significant concentrations of 13 PAHs were found in 40 samples of different vegetable oils from Iran’s market, including frying, blended, sunflower, corn, and canola oils. The mean values for BaP, PAH4, PAH8 (PAH4 +BkF + BghiP + DbahA + IP), and PAH13 were 1.91, 12.50, 19.60 and 35.54 μg/kg, respectively. Altogether 30 and 35% of the evaluated samples exceeded the current EU maximum permitted levels [66]. Tea, the most globally consumed beverage processed from tea plant leaves, is susceptible to accumulating high levels PAHs due to its high surface area. Moreover, the manufacturing process of tea leaves, such as the drying step that involves the utilization of combustion gases from burning wood, oil, or coal, can also lead to the generation of PAHs. Previous studies have reported high levels of PAHs in tea leaves [67,68]. According to Gao et al. [69], PAHs were detected in tea with a detection rate close to 100% globally over the past two decades and the contents ranged from 100 to 10,000 μg/kg, with black tea displaying higher levels compared to dark tea and green tea. Although tea leaves are commonly used for preparing infusions rather than direct consumption, it should be noted that the release of PAHs from tea increases with infusion time [68,70], and therefore, it is advisable to minimize this time for teas that contain high levels of PAHs.

Studies have shown the levels of PAHs in various food samples from different countries, as summarized in Table 3. It is evident from the table that food is contaminated with PAHs to varying degrees, which has become a global issue, and the contamination of PAHs is mainly concerned with the 16 EPA PAHs. The content of PAHs also differs among food types, with smoked foods being the most heavily contaminated. However, despite the high levels found, smoked foods do not contribute significantly to the overall human intake of PAHs, as they typically constitute a small portion of the diet. Furthermore, the PAH amounts in food vary by country; in China, Nigeria, Argentina, and Turkey, Argentinean tea was found to have the highest levels of PAHs ranging from 509.7 to 2746.5 μg/kg, while Nigerian tea had the lowest, ranging from 0.76 to 44.57 μg/kg. This suggests a serious pollution level of PAHs in Argentina. These discrepancies might stem from differences in industrial and economic development levels among countries. Given these findings and the potential health risks associated with PAHs, their presence in food should be given more attention as persistent pollutants.

## 5. PAH Detection in Food

### 5.1. Sample Preparation

**Figure 3:**
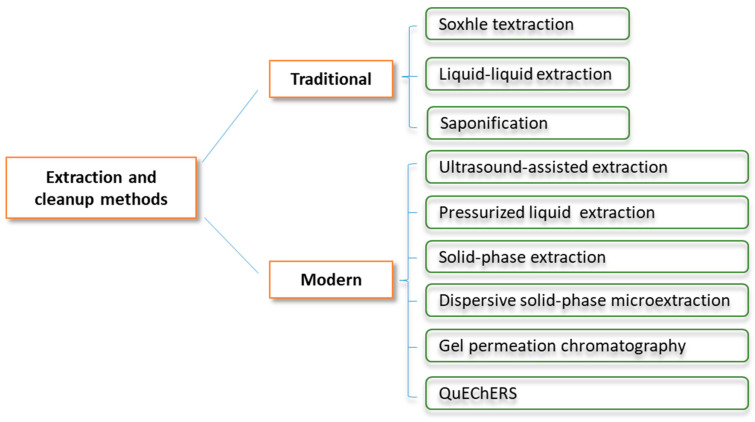
Different extraction and cleanup methods used for PAH analysis in food.

Sample preparation is critical for achieving accurate results in PAH analysis since PAH contents are extremely low and can be strongly influenced by the components of the food matrix. The protocol generally involves two main steps: (i) extracting PAHs from food matrices employing an appropriate method and (ii) purifying the extracts to remove undesired compounds. The choice of suitable extraction and cleanup procedures is paramount to ensure efficient isolation and purification of the targets, while concurrently reducing matrix effects and interferents. Ideally, a sample preparation method is supposed to be a simple process that offers high efficiency while minimizing time and cost. Different extraction and cleanup methods for PAH analysis are represented in Figure 3.

During sample preparation, all the utensils applied in the analysis should be meticulously cleaned with high-purity acetone or hexane prior to use, thereby ensuring that any detected PAHs are not sourced from the equipment itself. To mitigate cross-contamination, it is advisable to utilize only inert materials, such as stainless steel, glass, or aluminum, when preparing the samples under examination. Plastic materials, especially low-density polyethylene, should be strictly avoided due to the potential for PAH adsorption. The exposure to high temperature, light, and smoke should be avoided as well since this can induce volatilization and chemical conversion. Furthermore, the duration of sample storage should be minimized to avert undesirable reactions, such as oxidation and decomposition, which may occur between the PAHs and the food matrix, potentially yielding hazardous compounds [16,104].

#### 5.1.1. Extraction Methods

A wide range of techniques, for instance, Soxhlet extraction (SOX) have been exploited for PAH analysis in diverse food items [57,87,105,106]. The SOX method is designed according to repeated solvent reflux and solvent siphoning that allows continuous interaction between the solvent and the sample and thus results in high extraction yield. However, Soxhlet extractors are usually intricate pieces of glassware, making them both fragile and relatively expensive. Moreover, this technique is often very time-consuming, typically requiring 6 to 20 h [57,87,106,107], which greatly hinders the efficiency of sample extraction. In response to these drawbacks, alternative and more efficient methodologies, such as Ultrasound-Assisted Extraction (UAE), Pressurized Liquid Extraction (PLE), or QuEChERS (Q-quick, E-easy, C-cheap, E-effective, R-rugged, S-safe) method, have gained popularity. These technologies provide improved extraction efficiency and reduced extraction duration compared to SOX, making them appealing options for researchers and analysts in PAH analysis.

UAE has emerged as a promising approach for extracting PAHs from various food samples [71,72,76,77,78,82]. This does not require the use of high temperatures, which minimizes the risk of thermal degradation of PAHs. This helps preserve the integrity of the extracted PAHs and in turn ensures accurate analysis and reliable results. During UAE, the sample and the solvent mixture are introduced into an ultrasonic device. The powerful vibration, fragmentation, and cavitation effects generated by ultrasonic waves facilitate the dissolution and diffusion of analytes in the sample resulting in effective extraction [108]. The primary advantage of UAE is the reduction in extraction time due to increased mass transfer, i.e., from 20 h with SOX to approximately 30 min with UAE [109]. The reduced extraction time was also noted by Zhu et al. who used UAE to extract 16 EPA PAHs from fried squid using *n*-hexane as the solvent. Good extraction yields with recoveries above 96% were obtained after 40 min of extraction at 30 °C with a sample–solvent ratio of 1:9 [110]. Selection of the solvent is one of the key factors in a successful UAE, and it depends primarily on the nature of the food matrix and PAHs to be extracted. Commonly used extraction solvents mostly include acetonitrile, acetone, *n*-hexane, dichloromethane, or their mixtures. Nevertheless, these solvents are both expensive and environmentally unfriendly due to their high toxicity. With the rising focus on green chemistry, efforts are being made to replace these solvents with more environmentally benign alternatives, and the most recent developments in this method are focusing on reducing the volume of solvents used. As an example, a recent study by Yang et al. [111] proposed an optimized UAE method that utilizes a supramolecular solvent composed of 1-octanol and tetrabutylammonium bromide, with the aim of analyzing BaP from fried and baked foods. In this study, 200 mg of dried samples was mixed with 600 μL supramolecular solvent to extract BaP. Notably, no evaporation or further cleanup steps were required for the extracts. The overall sample treatment time was approximately 30 min with recovery rates ranging from 89.86 to 100.01% and a limit of detection (LOD) of 0.11 μg/kg. The supramolecular solvent used in this strategy exhibited low toxicity and only small amount usage that aligns with the principles of green chemistry [112], demonstrating promising prospects for the wide range of applications in the future.

**Figure 4:**
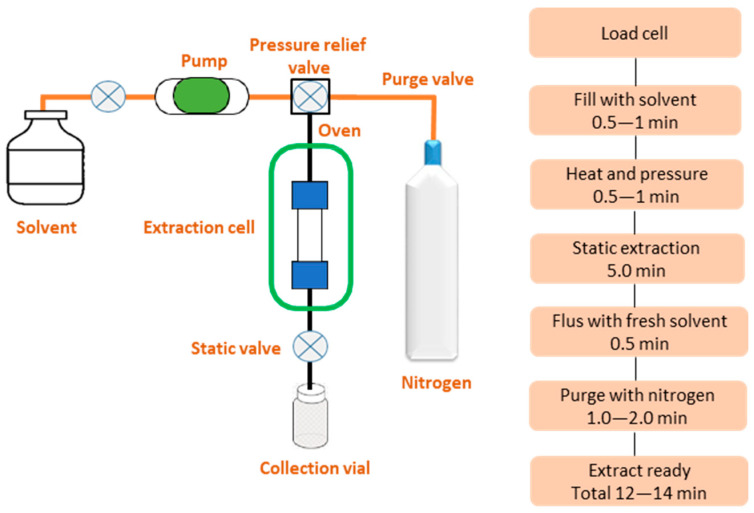
Schematic diagram of a PLE system.

PLE, also known as Accelerated Solvent Extraction (ASE), employs a solvent at elevated temperature (*Textr*) and pressure (*Pextr*) to keep the solvent in the liquid state for the extraction of PAHs primarily from solid food samples. This extraction involves placing the sample in a pressurized vessel along with a solvent such as *n*-hexane and heating it to extract the analytes from the matrix. *Textr* and *Pextr* are two key parameters to be optimized when performing PLE [113]. *Textr* enhances the efficiency of extraction due to improvement in the diffusion rate and solubility of analytes in solvents. However, it may also increase analyte degradation, especially when combined with prolonged extraction times. In addition to that, the solubility of matrix components also increases with temperature, producing more interferences that may affect the accuracy of subsequent determination. *Textr* typically varies between 50 and 200 °C and is dependent on the target analyte. On the other hand, *Pextr* is used to keep the solvent below its boiling point, facilitating its penetration into the matrix pores. In PLE, *Pextr* usually varies from 5.0 to 20 Mpa [114]. These *Textr* and *Pextr* conditions result in high extraction yields of the analytes while reducing both time and solvent usage. More recently, Suranová et al. [115] proposed a PLE-based method to extract PAH4 from smoked meat products. In this study, the sample was mixed with silica gel as a dispersing agent and then extracted with *n*-hexane at 100 °C and 10 Mpa for three static cycles of 10 min each, which provided almost 100% recovery. Pissinatti et al. [116] established a technique combining PLE with liquid–liquid extraction (LLE) to extract 10 PAHs from roasted coffee. The PLE method was operated using a hexane/dichloromethane (85/15, *v*/*v*) extraction solution at 100 °C and 10.34 Mpa with two cycles of 5 min each, which yielded satisfied results with recoveries ranging between 87 and 111%. PLE can also be easily automated, and commercial PLE systems can extract up to 24 samples simultaneously without the need for human intervention. This reduces reliance on manual labor and minimizes the potential for human error. However, PLE requires specialized equipment that can withstand high temperatures and pressures, making it expensive and inaccessible for many laboratories, which is a disadvantage of PLE [117]. A schematic diagram of a PLE system is shown in Figure 4.

**Figure 5:**
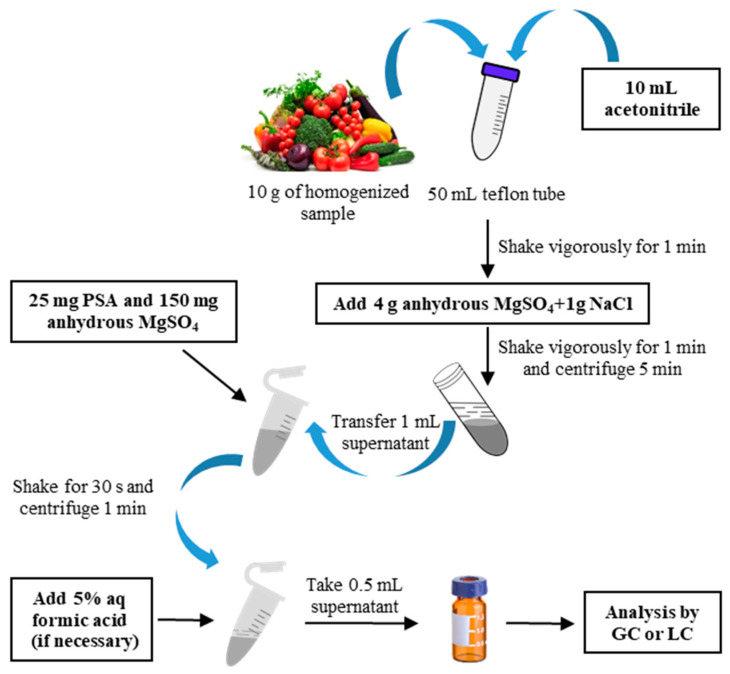
Schematic of the main steps of the original QuEChERS analysis technique [122].

The methods for extracting PAHs, such as SOX and UAE, are generally both cumbersome and expensive. As a result, the QuEChERS method has been extensively used in PAH analysis in recent years [118,119,120,121]. Figure 5 presents a simplified diagram of the QuEChERS extraction originally established for vegetable and fruit samples with high moisture content [122]. This technique combines extraction, isolation, and purification in a single run and offers flexibility by selecting different solvents, salts, and buffers for salting-out partitioning, as well as employing varied sorbents for the dispersive SPE (d-SPE) process. It demonstrates the merits of simplicity, convenience, time and cost savings, and low solvent consumption but high recovery rates for the PAHs [123]. Since the food matrices are diverse and complex, the extraction conditions are also different and must be carefully assessed when employing this methodology. In most cases, various extraction solvents have been investigated to optimize the process to match the matrix. To determine 16 EU priority PAHs from six categories of foodstuffs, Chiang et al. [124] developed a QuEChERS method and compared the extraction efficiency of acetonitrile, acetonitrile with 1% acetic acid (*v*/*v*), acetone, and acetone with 1% acetic acid (*v*/*v*). It was found that different solvents should be applied to different food matrices to extract these PAHs. Acetone had a higher extraction efficiency for protein-based matrix categories (e.g., poultry and meat, fish and seafood, and soybeans and products), whereas acetonitrile with 1% acetic acid (*v*/*v*) had a higher extraction efficiency for carbohydrate-based matrix categories (e.g., grains, root vegetables, and coffee). Using this method, the rapid extraction of targets from diverse food matrices was achieved within 30 min with recovery rates being 65–111% at optimized conditions. In a separate study, a comparable technique was utilized to extract 16 EU PAHs from charcoal-grilled chicken drumsticks with acetone identified as the best extractant. In the subsequent analysis of samples using high-performance liquid chromatography (HPLC) diode array detector (DAD) and fluorescence detector (FLD), the recovery values were found between 67 and 114%, and limits of detection (LODs) and quantification (LOQs) were 0.004–0.25 and 0.01–0.75 μg/kg, respectively [125]. In addition, researchers have made innovations in the selection of sorbents such as the application of emerging sorbents to suit more complex samples with superior cleanup efficiency. Edible oils are known to be a troublesome matrix as they mainly comprise various fats, and PAHs generally have a similar polarity to the oil matrix, which poses a challenge in PAH analysis in oily matrices. Zacs et al. [126] developed a QuEChERS technique combined with gas chromatography–tandem mass spectrometry (GC-MS/MS) for the quantification of PAH4 in edible oils. They utilized multiwalled carbon nanotubes (MWCNTs) as sorbents to effectively eliminate interferents in the samples, which provide acceptable purity of the final extracts and good accuracy of PAHs. The LODs and LOQs were found over the range of 0.06–0.21 and 0.1–0.71 μg/kg, respectively, while the recovery was 96–107%. Despite the satisfactory results achieved with the QuEChERS technique, there have been instances where it has been proven to be insufficient for purifying analytes in the case of very complex food matrix. Accordingly, a combination of other treatment techniques is needed for efficient sample cleanup [127]. In this regard, Agus et al. [128] combined with dispersive liquid–liquid micro-extraction (DLLME) to extract PAH4 from roasted cocoa beans after the QuEChERS methodology was performed using acetonitrile as the extractant, MgSO_4_ and NaCl as salting-out agents, and primary secondary amine (PSA) and octadecylsilane (C18) as cleaning sorbents. Low LODs and LOQs (0.02–0.08 and 0.08–0.30 μg/kg, respectively) and recoveries between 52.1 and 91.6% were achieved, which complied with the limit (50–120%) set by the EU.

Apart from the aforementioned methods, several other techniques such as saponification and LLE are also employed for the extraction of PAHs from food samples as alternatives in laboratories. Owing to the lipophilic character, the isolation of PAHs from lipids in oil- and fat-rich foods must be performed to ensure complete extraction. Saponification of lipids is a common approach for separating PAHs from such food samples [129,130,131]. This process involves the use of methanolic or ethanolic KOH or NaOH solution to hydrolyze oils and fats into fatty acid salts, which can then be removed. To determine PAH4 in vegetable oil samples, Zachara et al. [132] developed a saponification method using a methanolic KOH solution coupled with an LLE procedure with cyclohexane as an extractant and a subsequent chromatography column packed with alumina prior to HPLC-FLD analysis. The LODs and LOQs were 0.18 and 0.25 μg/kg, respectively, and the recoveries were 83.42–108.65%. In a different study, they adopted a similar method successfully to analyze PAHs in smoked meat and fish products, and good results were obtained [133]. For the extraction of PAH4 from fish and shellfish matrix, a saponification reaction of the samples with an ethanolic KOH solution was also proposed as a preliminary step to LLE. The method yielded recoveries from 77 to 108% with LOQs in the range of 0.06–0.26 μg/kg [134]. However, it should be noted that the use of strong alkali may result in the degradation of certain PAHs, such as Acen, due to its alkali-sensitive nature [135]. In addition, Martinez et al. [136] have reported instances of BaP losses due to partial partitioning into the alcoholic phase during saponification. LLE performs the separation of compounds primarily based on their relative solubilities in immiscible liquids. In the case of liquid food matrices such as oil and tea infusion [132,137,138], LLE is a well-recognized extraction technique due to its reliability, versatility, and compatibility with a wide range of instruments. A study by Girelli et al. [139] applied an LLE method with cyclohexane following saponification to analyze 15 PAHs in tea infusions via HPLC-FLD. The recoveries of most PAHs were found to be satisfactory (70–130%) with the exception of Ace and Fluo, which present low recoveries (<50%) when the spiked level was below 2 ng. They also compared LLE and UAE methods and found that LLE outperformed UAE in terms of extracting PAHs from tea infusions, as it required smaller sample volumes and achieved higher recovery rates. Despite that, the major drawbacks of LLE include difficulty to automate, being a time-consuming, laborious, and solvent-intensive process that is not environmentally friendly, and lack of specificity towards target analytes, which cause high matrix effects.

#### 5.1.2. Cleanup Procedures

The crude extracts of food samples usually contain co-extracted components, such as oils, lipids, waxes, and pigments, which can adversely affect identification and quantification of PAHs. As a result, for obtaining better-quality extracts, a cleanup step is vital to remove these unwanted compounds after extraction and prior to detection. The protocols described for the cleanup of PAHs in foods mostly include column chromatography, Solid-phase Extraction (SPE), Gel permeation Chromatography (GPC), and Solid-phase Micro-extraction (SPME).

In column chromatography, purification was achieved by placing the sample solution containing PAHs onto a glass chromatographic column packed with specific solid sorbents that have been preconditioned and activated, followed by elution with proper organic solvents. The suitable sorbents are the guarantee for obtaining high cleanup performance and recovery rate. Silica gel, alumina, Florisil, and C18 are commonly utilized sorbents, wherein silica gel is the most prevalent due to good adsorption capacity, low cost, and easy availability [76,87,90,140,141]. For example, Hossain et al. [140] proposed a methodology for the analysis of eight PAHs in edible oils (e.g., soybean oil, mustard oil, and coconut oil). Cleanup of the crude extracts was performed using silica gel column chromatography followed by elution with an acetonitrile/acetone solution (60/40, *v*/*v*) before GC-MS analysis. Similarly, a technique was developed by Guatemala-Morales et al. [141] to purify 16 PAHs from roasted coffee. The cleanup was performed by pairing the alkaline saponification step together with a silica gel column chromatography. In some cases, to obtain better performance, two or more sorbents were used together in the cleanup process. Li et al. [142], for instance, utilized GPC and column chromatography packed with silica gel and neutral alumina, followed by elution with dichloromethane/*n*-hexane (1/1, *v*/*v*) and GC-MS/MS to evaluate 16 EPA PAHs in coral reef fish. In another study, Fang et al. [143] adopted C18 and Florisil chromatographic bi-column for cleanup using acetonitrile as the elution solution and liquid chromatography–tandem mass spectrometry (LC-MS/MS) to analyze eight PAHs in aquatic products. All PAHs’ recoveries were over 86% with relative standard deviations (RSDs) < 4.0%. Though column chromatography methods yield satisfactory results; however, they do require large volumes of organic solvents during column precondition, rinsing, and elution, and result in significant quantity of waste. Due to this, they are not considered environmentally friendly.

**Figure 6:**
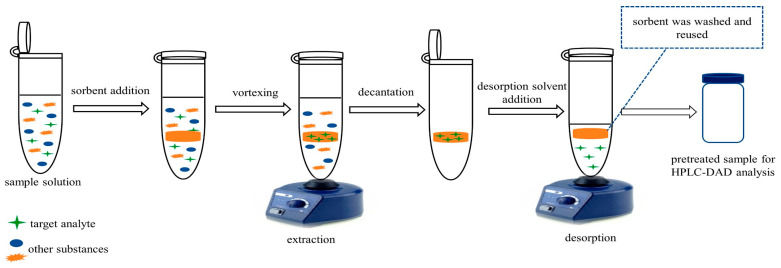
Illustration of the main steps of the VA-SPE procedure [151].

SPE is based on the utilization of the sorbent material to selectively bind and release PAHs under specific conditions [144,145,146]. As a result, the choice of the sorbent is very important. When performing an SPE, the sorbent is preconditioned and activated using an organic solvent. Sample solution is then passed through it, where analytes are retained, while unwanted matrix components are washed out. By eluting with other organic solvents, pure analytes can be obtained. SPE sorbents are usually packed into cartridges or micro-columns and are typically of powder shape [147]. A typical SPE is carried out to cleanup or isolate PAHs from food samples using silica gel, Florisil, and alumina [89,92,93]. Xu et al. [148] compared these three sorbents for extraction and isolation of 16 EPA PAHs. Florisil was observed to induce significant degradation of certain PAHs (mainly BaP) and result in low recovery rates (<27.1%). Alumina exhibited strong adsorption to PAHs, rendering it unsuitable for sample cleanup. In contrast, silica gel showed satisfactory efficacy in both clean extracts and PAH recovery, making it the preferred choice for PAH analysis. However, some drawbacks such as high expense and non-reusability restrict their long-term application. This led to an extensive research activity for exploring new SPE-based technologies or/and novel sorbents with exceptional extraction performance. As an example, Nazir et al. [149] introduced a novel micro-SPE (μ-SPE) method utilizing spent tea leaves (STLs) as a sorbent and applied it successfully to water, rice, apple, and orange juice samples for the extraction of five PAHs, namely, Flu, Fluo, Pyr, Chr, and BaP. STLs, a byproduct of the tea brewing process, are rich in lignin, cellulose, hemicellulose, polyphenols, and aliphatic carbon, which enable the adsorption of PAHs and can extract PAHs from selected matrices through π-π interaction with a good percentage recovery. Samples spiked with PAHs recovered above 88.0% with RSDs less than 9.8%. The appealing features of this biomaterial such as low cost and the absence of any modification requirements are expected to create new avenues for the vast utilization of agricultural waste. In another study, Asfaram et al. [150] proposed a magnetic dispersive micro-solid phase extraction (MD-μ-SPE) method using a magnetic Cu:CuO–graphene oxide nanocomposite as the sorbent, which has good extraction performance for four PAHs, namely, Nap, Phe, Ant, and Pyr, with enrichment factors ranging from 116.51 to 133.05. Combined with HPLC-UV, they achieved the accurate monitoring of these PAHs in real samples, including vegetables, fruits, and environmental water samples. The method exhibited LODs and LOQs ranging from 0.01to 0.061 and from 0.485 to 2.034 ng/mL, respectively, with recoveries between 95.1 and 106.8% and RSDs < 5.62%. Zaw et al. [151] prepared a gelatin aerogel tablet and used it as a vortex-assisted SPE (VA-SPE) sorbent for the extraction of PAHs (BaA, BbF, and BaP) from tea infusions before detection via HPLC-DAD (as illustrated in Figure 6). This tablet is easy to synthesize with good reproducibility (RSDs < 0.24%, *n* = 6) and excellent reusability (*n* = 40, RSDs < 0.17%), making it more flexible and low-cost (USD 0.0004 per tablet). By adopting the VA-SPE method and eluting with 2.5 mL of hexane, the analytes were efficiently extracted within 1.5 min, and lower LODs at 1.65–2.06 ng/L and recoveries between 70.1 and 119.3% were achieved. In comparison with other methods, this technique is simpler and more convenient and requires shorter extraction time and smaller sample volume. In another study by Wang et al. [152], they synthesized a β-cyclodextrin functionalized graphene oxide-grafted silica (β-CD/GO/SiO_2_) sorbent, which exhibited high extraction efficiency for PAHs with 4- to 5-ring structures, such as BaP, BbF, and BaA. They obtained satisfactory recovery rates (91.2–109.1%) and LODs (0.1–0.3 μg/L) using β-CD/GO/SiO_2_ SPE cartridge and eluting with 1.95 mL of acetone to extract PAHs (BaP, BbF, and BaA) from fried chicken. Comparatively, this method offers lower or comparable LODs and wider or comparable linear ranges, possibly due to the strong π–π stacking, hydrophobic interaction, and size complementarity between β-CD/GO/SiO_2_ and the analytes. Molecular imprinting polymers (MIPs) are also applied in SPE. They present high adsorption capacity, good reusability, and remarkable selectivity because of their template-based synthesis against the analyte [153]. Using MIP-based SPE methods, PAHs in kebabs, oil–tea camellia seed oil, and tea have been thoroughly researched [145,146,154,155]. Although SPE techniques are effective at purifying or separating PAHs from diverse food matrices, they do have some drawbacks. For instance, the clogging of cartridges by sample suspended matter can lead to reduced recovery rates due to interactions between the sorbents and analytes. Furthermore, commercial SPE columns are not reusable, which may increase experimental costs.

**Figure 7:**
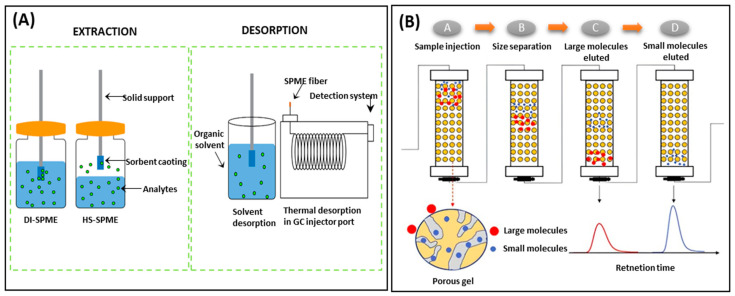
(**A**) General scheme for the extraction and desorption steps in SPME analysis [157]. (**B**) Schematic diagram of the separation principle of GPC test [164].

SPME evolved from SPE, an appealing approach that necessitates minimal or no solvent and only small volumes/amounts of sample. So far, the SPME applied in the detection of PAHs in food samples were mainly operated in direct immersion (DI-SPME) or headspace (HS-SPME) modes [156]. Figure 7A presents a general scheme for the extraction and desorption steps in SPME [157]. This extraction relies on the partitioning of analytes between the coating of SPME fiber and the sample matrix, followed by the desorption of the analytes into an appropriate instrument by the provision of heat or application of a desorption solvent. As a result, the choice of suitable fiber coating is of prime importance in SPME applications [158]. Commercially available fiber coatings like polyacrylate (PA), polydimethylsiloxane (PDMS), carboxen/PDMS (CAR/PDMS), and divinylbenzene/CAR/PDMS (DVB/CAR/PDMS) are preferred for the extraction of PAHs from food products because they provide adequate sensitivity and good recoveries. Very recently, Aresta et al. [159] applied DI-SPME using a 85 μm PA fiber to effectively extract nine PAHs from coffee samples, such as ground coffee, infusion, and coffee grounds. The PAH contents were later determined using GC-MS. The procedure is eco-friendly and eliminates the need for further pretreatment of the sample, except simple water dilution. The LODs of the method ranged between 0.3 and 16.6 ng/g with recoveries between 59 and 99%. Fathollahy et al. [160] utilized a PDMS-coated SPME fiber (60 μm) to successfully extract eight PAHs from edible oils. After extraction, the analytes were desorbed and analyzed by GC-flame ionization detector (FID). Despite the increasing applications of these fiber coatings, they have limitations such as poor mechanical strength, fragility, and short lifetime; therefore, the development of novel and improved coating materials has emerged as a prominent research focus in the field of SPME technology. Erdem et al. [161] recently prepared an SPME fiber by coating clay–chitosan and dicationic ionic liquid onto a stainless steel wire. This novel fiber demonstrated extraction recoveries from 87.5 to 112% with LODs at 0.1–10 ng/L in both HS and DI-SPME methods for the analysis of 16 EPA PAHs in coffee and tea samples. As compared to commercial SPME fibers like CAR/PDMS and DVB/CAR/PDMS, this innovative fiber offers benefits such as easy preparation, low cost, and improved extraction efficiency. Moreover, it can be reused over 125 times while maintaining stability. Zhao et al. [162] developed a novel covalent organic framework/MXene (COF/MXene) composite and applied it as a HS-SPME coating combined with GC-FID to analyze five PAHs (Acen, Phen, Flu, Ant, and Pyr) in honey samples. The results demonstrated low LODs (0.2–0.6 ng/g), satisfied recoveries (73.2–112%), and great precision (≤9.40%). The COF/MXene fiber also provides high enhancement factors (483–598) and a long lifetime (300 cycles of use), highlighting its impressive chemical stability and potential to reduce experimental costs. Furthermore, Xu et al. [163] introduced a novel MIL-88-NH_2_/COF composite coated on stainless steel wires as an HS-SPME fiber in the detection of five PAHs (Nap, Acen, Aceny, Phen, and Flu) in tea samples by GC-FID. The LODs of the selected PAHs were obtained in the range of 0.019–0.023 ng/mL. The MIL-88-NH_2_/COF-coated fiber exhibited good durability (100 cycles of use), excellent extraction efficiency (51.70–103.64%), and high adsorption capacity with enhancement factor up to 9858. These new SPME coatings are very advantageous for the extraction of PAHs from different food matrices; however, the majority of such coating materials remain in the experimental and research phase, which limit their commercial availability and widespread utilization.

GPC, also referred to as size-exclusion chromatography, is a highly effective post-extraction cleanup technique for removing interferences with big molecules (e.g., lipids, proteins, and pigments). It provides separation based on differences in molecular size, with larger molecules eluting first, then smaller ones (Figure 7B) [164]. The separation process occurs in a glass column that is packed with stationary phase materials such as styrene–divinylbenzene copolymer (i.e., Bio Beads SX-3) [85,165]. In a study conducted by Wang et al. [166], GPC was applied after UAE extraction to purify oil extracts with the aim of analyzing 14 PAHs in edible oils by HPLC-DAD-FLD. By utilizing Bio Beads SX-3, the GPC procedure effectively eliminated triglycerides from the samples, achieving method recoveries ranging from 73 to 110% and RSDs < 10%. Traditional GPC systems suffer from low sample loading capacity (about 0.15 g) and limited cleanup ability, especially when treating samples high in fat content. To further improve the detection of PAH4 in olive oil samples, Cotugno et al. [167] developed a novel double-column GPC system. The system uses two glass chromatographic columns and a switching valve, allowing for the removal of interfering analytes in olive oil without resorting to any preliminary extraction process. The system enables a sample loading capacity up to 1 g, as well as improved LODs and LOQs in the range of 0.21–0.32 and 0.70–1.06 ng/g, respectively. Moreover, the possibility to process up to 15 samples in a single GPC sequence and the automatization of the chromatographic separation make this technique more appealing. Rozentale et al. [85] used a Bio Beads SX-3 gel permeation column and a Silica SPE column to successfully remove co-extractives in the cleanup of PAH4 in cereal products with high protein and fat content. LODs of 0.002–0.006 μg/kg and recovery rates between 92 and 117% were obtained. The drawbacks of GPC include the requirement for big columns, extended operation times, and high cost due to the significant amount of solvents needed for eluting.

### 5.2. Detection Strategies

Due to the multiple types and varied structures of PAHs, as well as the diversity of food matrices, the detection of PAHs should take into account several factors like the specific application, the nature of PAHs, and the type of samples being analyzed. In recent years, the most commonly used strategies for detection and quantification of PAHs in foodstuffs are GC and LC, primarily because of their exceptional sensitivity, separation capabilities, and identification prowess. Each of these techniques offers unique advantages and constraints, with factors including sensitivity, selectivity, and sample preparation influencing the choice of method. Table 4 summarizes GC- and LC-based techniques recently developed for PAH determination in various food samples.

#### 5.2.1. Gas Chromatography

GC is a popular chromatographic technique for PAHs’ detection in food matrices since PAHs are thermally stable and the majority are volatile and readily vaporized. PAH analyses using GC are accompanied by detectors such as MS [159,161,168,169,170,171,172,173], MS/MS [154,174,175,176], FID [121,160,162,163], or high-resolution MS (HRMS) [57,85,177] Nevertheless, more recent studies tend to utilize MS combined with GC to detect PAHs in food as it offers better method sensitivity, selectivity, and identification abilities compared to FID, and is more convenient and cost-effective than MS/MS and HRMS. In some nations, GC-MS is a component of national standard methods for the determination of PAHs in different food categories (e.g., cereals/cereal products, meats/meat products, and aquatic foods/aquatic food products) [178].

When conducting GC-MS analysis, the choice of an appropriate injection mode is crucial to avoid discrimination, especially with less volatile compounds, such as PAHs. The most utilized injection modes in PAH analysis are splitless [161,168,169,170] and programmed-temperature vaporization (PTV) [179,180] injection. Splitless injection typically allows for a small sample injection capacity of up to 2 μL and retains non-volatile co-injected chemicals in the liner, which limits sensitivity for complex food matrices. On the other hand, PTV injection enables larger sample sizes up to 5 μL and releases high-boiling co-extracted chemicals through a split vent or traps them on a liner to reduce matrix effects; as a result, it is extensively employed to minimize injection discrimination and enhance sensitivity in food samples [181]. Electron ionization (EI) is a “hard” ionization technique that results in the reproducible fragmentation of PAHs into well-characterized mass spectral fingerprints valuable for qualitative identification. However, due to the high stability of PAHs, the use of EI produces mainly the molecular ion ([M–H]^+^ or [M–2H]^+^) with few fragments, and thus, the single quadrupole analyzer is often employed [168,169,172,182,183]. To accomplish the valuable identification and quantification of the PAHs being analyzed, it is essential to select at least 2–3 ions for each analyte. The selected ion monitoring (SIM) is used where the analyte mass-to-charge ratio (*m*/*z*) is concentrated to achieve a lower LOD and LOQ with reduced interference. Nevertheless, in the case of several groups of isomeric PAHs, such as Ant and Phen, BaA and Chr, and BbF and BkF, their chromatographic peaks usually overlap, and the MS fragment ions and relative abundances are alike, which makes GC-MS unable to accurately identify and quantify such compounds [184]. Improving GC isolation is one solution. In fact, to a large extent, the presence of unresolved isomers is attributed to the stationary phase used; thus, the utilization of a suitable column can help solve such an issue. It is recommended to use nonpolar stationary phase consisting of 5% phenyl and 95% dimethylpolysiloxane, which is recognized as the universal choice for PAH analysis column [109,141,146,168,183]. Furthermore, MS/MS or HRMS detection’s superior selectivity and sensitivity allowed it to identify and quantify these PAHs that were poorly differentiated by GC through the use of multiple reaction monitoring and accurate masses of fragment ions. It is worth noting that these methods are expensive and highly specialized and have high operating and maintenance costs, which makes them unavailable in most laboratories.

In general, the GC-MS system more often used consists of a split/splitless injector or a PTV injector, a capillary column such as HP-5MS or DB-5MS, an EI source, and a quadrupole (Q) analyzer that operates in the SIM mode. Recent advances in injection systems primarily involve the use of thermal desorption (TD). TD units function by heating the extracts or sorbents that retain the analytes in a flow of inert carrier gas, thereby releasing the retained analytes into the analytical system [185]. With the coupling of on-line dynamic headspace extraction, this system has been applied to determine six PAHs (Nap, Acen, Aceny, Flu, Phen, and Ant) in fish products. The LOQs were 0.01–0.6 ng/g and absolute recoveries were 13–62%. The method achieved the detection of these PAHs in a wide range of seafoods with concentrations from 0.3 to 26.2 ng/g [186]. Furthermore, TD has also shown excellent performance in the determination of PAHs and nitro-PAHs in PM10 and PM2.5 [187]. Developments in separation have been observed from the use of novel columns or/and comprehensive two-dimensional GC (GCxGC) coupled with MS or TOF-MS. GCxGC offers advantages such as higher peak capacity and better resolution and separation of various groups of PAHs and their derivatives compared to one-dimensional GC. As an example, using a GCxGC-TOF-MS system, a total of 97 PAHs, including parent, alkyl-, nitro-, oxy-, thio-, chloro-, bromo-, and high-molecular-weight PAHs, were separated and detected in a 176-min run [188]. In another study, GCxGC-MS was employed to the analysis of 15 PAHs in seafood including fish and mussel [189]. Recently, some research endeavors have advanced the development of an HPLC/GC-MS system for the analysis of PAHs [190]. In this setup, the HPLC apparatus is employed to separate PAHs from the main components of food matrices using backflush fractionation. This integrated chromatographic system facilitates automated sample cleanup with HPLC and large volume injections on the GC, thereby enhancing detection capabilities compared to conventional GC/MS analysis. Advances in mass analyzers have also been reported. Q-Orbitrap-MS, IT-MS, and Q-time of flight MS (Q-TOF-MS) have provided accurate quantification coupled with GC. A state-of-the-art technology in the field is a proposed isotope-dilution GC coupled with HRMS (ID-GC-HRMS) for the determination of four PAHs (BaA, Chr, BbF, and BaP) in olive oil. Satisfactory recoveries ranged from 97.5 to 102%, RSDs were <1%, and measurement uncertainty was <5%, confirming that the method is suitable for the certification of PAHs in olive oil certified reference materials [191]. Prata et al. [118] explored a GC-Q-Orbitrap-MS method with QuEChERS-based extraction to evaluate 14 PAHs and 4 pesticides in baby food samples commercialized in Brazil. For PAHs, recoveries between 71 and 120% were obtained, and high sensitivity was achieved with LODs at 0.05 μg/kg for the different compounds. To investigate honey contamination, a cheap and multiresidue method was developed for the analysis of 16 PAHs, 90 pesticides, and 22 polychlorinated biphenyls [192]. In this methodology, GC-IT-MS/MS was used to detect PAHs in honey matrices after QuEChERS and SPME extraction. The LODs were between 0.07 and 12 ng/g, and the LOQs were between 0.23 and 40 ng/g. Nine PAHs were found in the majority of the honey samples from Lebanon, with concentrations varying from 5.36 to 33.3 ng/g. The validation of GC–IT/MS with ultrasound–vortex-assisted DLLME demonstrated enhanced sensitivity for detecting nine PAHs in honey matrices with LODs of 0.030–0.199 ng/g, leading to a 316–1200-fold higher sensitivity compared to common GC-FID [179]. Additionally, GC-Q-TOF-MS combined with appropriate sample preparation techniques such as QuEChERS has been utilized for the detection of PAH4 in fish and malt with LOQs in the range of 0.12–0.24 μg/kg [193].

GC-FID has poor selectivity and low sensitivity, in comparison to MS-related techniques, but it is inexpensive and easy to use, and a wealth of literature is still available on the application of this method for analyzing PAHs in various types of food samples by combining with efficient sample preparation methods. Recent examples of GC–FID used to determine PAHs in food matrices include edible oils, honey, tea, etc. [121,160,162,163].

#### 5.2.2. Liquid Chromatography

The LC-based method has long been utilized for the quantification of PAHs in food and also in some official methods proposed by nations such as China [178,194] and the US [195]. LC detection does not have to take into account PAHs’ volatility or molecular weight since it operates at lower temperatures. Thermal breakdown of heavy PAHs is another advantage offered by LC. When analyzing PAHs, LC can be utilized with FLD [132,142,196,197,198], DAD [116,151,152], or UV [73,150,199] detector, wherein FLD is more precise and sensitive than UV or DAD. Chiang et al. [125] developed a methodology for determining 16 EU priority PAHs in charcoal-grilled chicken drumsticks. They compared three major PAHs analytical methods, with HPLC-FLD being the most appropriate method because of its superior sensitivity and shorter analysis time than GC-MS and HPLC-DAD. Nevertheless, the use of DAD or UV is still a necessity for the detection of non-fluorescent PAHs like cyclopenta[c, d]pyrene. In LC, another important factor is the choice of column. In general, silica-based columns containing C18 with varying lengths and diameters are the most employed for the separation of PAHs, and to decrease the runtime, the gradient mode has been used with acetonitrile or methanol gradient in water [132,152,198,200]. In some cases, determinations are conducted in the isocratic mode, and detector combinations, such as UV and/or DAD coupled with FLD [73,145,201], are also employed. For instance, Singh et al. [200] established a HPLC-FLD method to analyze 16 PAHs in cooked chicken and roasted coffee. The separation was achieved using a C18-ODS2 column and acetonitrile/water (90/10, *v*/*v*) in the isocratic mode, providing high sensitivity with LODs of 0.03–0.06 ng/mL. To investigate the concentration, source, and risk of 22 PAHs from Chinese tea products, Guo et al. [72] proposed an analytical methodology employing HPLC-DAD-FLD and gradient elution with water and acetonitrile. LODs of the method were 0.10–0.75 μg/kg, and the total concentration of Σ22 PAHs in 28 varieties of 119 tea samples varied from 136.99 to 51 μg/kg. Further, Hamidi et al. [201] presented a technique for evaluating the bioaccessibility of 15 PAHs in charcoal-grilled beef and chicken across different segments of human gastrointestinal tract. This was achieved through HPLC-UV-FLD analysis using gradient elution with acetonitrile in water. The method LODs were between 0.025 and 5 ng/g. The total PAHs were found in beef (30.73 ng/g) and chicken (70.93 ng/g) before its digestion. In addition to HPLC, ultra-high performance liquid chromatography (UHPLC) methods have also been proposed [166,202,203] coupled with FLD or/and DAD for PAH analysis in food examples (e.g., honey, edible oil, and coffee). Compared to HPLC, UHPLC allows for improved chromatographic resolution, increased throughput (total analysis time reduced by about 3/4-fold), and significantly reduced solvent consumption (by more than 70%), making it more environmentally friendly and cost-effective.

Despite the use of LC coupled with UV, FLD, and DAD systems, it remains challenging to offer structural information for the identification of PAHs in foods of different categories. The incorporation of an MS detection system in LC has emerged as a viable solution, and relevant determinations have been described in recent studies. More recently, Dueñas-Mas et al. [204] have reported a method for the estimation of four oxygenated PAHs (naphtacene-5,12-dione, 6H-benzo(cd)pyren-6-one, 11H-benzo(b)fluoren-11-one, and 9H-fluoren-9-one) in processed seafood, fish, and meat samples. This approach utilizes a rapid resolution LC-HRMS system equipped with a Q-TOF analyzer and an atmospheric pressure chemical ionization (APCI) source operating in the positive mode. LOQs of the method were in the range 0.08–4 ng/g. The total PAHs were detected at levels ranging from 3.5 to 12.6 ng/g, mainly in fish and seafood. In contrast to APCI, atmospheric pressure photoionization (APPI) demonstrates superior efficacy in ionizing nonpolar PAHs and their derivatives, while also exhibiting reduced ion suppression. Typically, to further increase the ionization a dopant (e.g., acetone or toluene) is employed. Lung et al. [205] have reported a multi-residue method for the analysis of 20 PAHs and 9 nitrated PAHs using UHPLC coupled with APPI-MS/MS using 0.5% 2,4-difluoroanisole in chlorobenzene as the dopant. The method offers the benefits of reduced separation time and superior resolution and sensitivity. The selected PAHs are separated in 15 min in the positive mode and 11 min in the negative mode, one half of GC-MS analysis time. LODs of the target PAHs, except acenaphthylene, and those of nitro-PAHs, except 2-nitrofluoranthene, are below 10 and 3 pg, respectively. To date, the application of LC-MS-based techniques to detect PAHs in food products is still limited, and this can primarily be attributed to the low ionization efficiency of PAHs, which is a result of their nonpolar characteristics.

#### 5.2.3. Other Technologies

Alongside the aforementioned methods, several other technologies such as enzyme-linked immunosorbent assay (ELISA) and surface-enhanced Raman spectroscopy (SERS) have also been developed to analyze PAHs in food samples. ELISA determines PAHs based on the principle of antigen–antibody interaction coupled with enzyme-catalyzed colorimetric changes, offering high sensitivity and selectivity. For PAHs, a type of small molecules, indirect competitive ELISA (ic-ELISA) is predominantly employed for their detection, with the pivotal aspect being the generation of antibodies capable of recognizing these compounds. Up to now, researchers have developed a range of anti-PAH antibodies using immunochemical techniques and established ELISA detection methods [206,207,208]. A study conducted by Wu et al. [209], they have prepared a highly sensitivity monoclonal antibody (mAb) and established an ic-ELISA method to detect Pyr and BaP in living aquatic products (e.g., fish, shrimp, and crab) with simple sample pretreatment. The method showed recoveries of 81.5–101.9% with LODs of 0.43–0.98 μg/kg and the coefficient of variation (CV) < 17%. Confirmation and relevance with HPLC-FLD has confirmed the ic-ELISA method as a feasible tool that lays a foundation for the development and application of PAH residue detection kit in aquatic products. In a separate study, Ma et al. [210] applied a streptavidin–horseradish peroxidase-based ELISA method utilizing synthesized polyclonal antibody (pAb) for the sensitive monitoring of BaP in environmental and food samples (e.g., vegetables, cereals, and barbecue food). This method achieved a low LOD of 0.0094 ng/mL and recoveries over the range of 91.12 to 109.23% with CV < 8.8%. With the 96-well microtiter plate, the analysis of nearly 100 samples can be completed within 6 h, indicating the rapidity and high throughput of this method. Moreover, Qiao et al. [17] synthesized an immunogen by conjugating 5-acenaphthylenecarboxylic acid to bovine serum albumin and produced a mAb with high affinities for Acen and Pyr. Based on this mAb, an ic-ELISA technique was developed for the sensitive detection of Acen and Pyr with a half-inhibitory concentration (IC_50_) of 12.17 ng/mL. However, this method has not yet been applied in food analysis. ELISA methods, on the other hand, require cumbersome incubation steps and repeated sample loading operations when handling multiple targets, affecting both convenience and detection efficiency.

SERS is emerging as a non-destructive, highly sensitive, and rapid detection technique that utilizes nanoscale precious metals such as gold (Au) or silver (Ag) as substrates. When the incident laser is resonantly coupled with the metal substrate at a certain excitation wavelength, a strong local electromagnetic field is formed, which is confined to a small range on the metal surface. This phenomenon known as local surface plasmon resonance (LSPR) leads to a significant enhancement of the Raman signals of the detected PAHs, thereby enabling highly sensitive identification and detection [211]. As an example, Su et al. [212] developed a liquid-interfacial SERS technology without pretreatment steps to achieve a rapid and direct detection of ultra-trace PAHs in edible oil. The sample and chloroform are miscible to form an organic phase. When gold nanoparticles (GNPs) are added and shaken vigorously, the GNPs reach the aqueous–organic interface and form a three-dimensional (3D) nano assembly. The characteristic peaks of edible oil and related molecules can be readily determined on a portable Raman spectrometer, with the entire detection process for one sample requiring only 3 min. The LOD of the method was as low as 0.1 μg/L. Wang et al. [18] utilized a SERS sensor composed of Au nanoparticles (AuNPs) and reoxidized graphene oxide (rGO) to simultaneously determine 16 EPA PAHs in Chinese traditional fried food (youtiao). The method involves depositing a higher density of AuNPs on the surface of rGO, resulting in a significantly reduced interparticle distance and the creation of more “hot spots.” LSPR from hot spots brings about greater enhancement of the PAH signal. The entire detection process is expedited, taking only 15 min, and the method LODs were estimated as low as 0.2–2 ng/mL. This method could prospectively be applied as a screening monitoring method to detect PAHs on-site for the quality control of fried food. However, this prepared sensor is performed under laboratory conditions during practical validation e.g., sample preparation and the complexity of sample types and matrices that is difficult to match in real-life situations, which presents a major obstacle for its practical application.

**Table 4:** A summary of GC- and LC-based methods for determining PAHs in food.

Sample	Analytes	Sample Pretreatment	Instrumental Techniques	Instrumental Details	Performance Characteristics	Ref.
Youtiao	16 PAHs	UAE with acetonitrile/acetone (3/2, *v*/*v*), purification by a C18 SPE cartridge and a Florisil SPE cartridge	GC-MS	DB-5MS capillary column (30 m × 0.25 mm i.d., 0.25 μm); programmed temperature; splitless injection; inlet, transfer line, and ion source temperature at 300 °C, 280 °C, and 230 °C, respectively	Recoveries: 72.2–108.1% LODs: 0.005–0.36 μg/kg	[71]
Tea	22 PAHs	UAE with *n*-hexane, purification by an SPE cartridge using carboxylated MWCNTs and diatomite as sorbents	HPLC-UV-FLD	C18 column (25 cm × 4.6 mm i.d., 5 μm) at 30 °C with a gradient mobile phase of acetonitrile and water	Recoveries: 82.1–97.4% RSDs: 2.3–5.9% LODs: 0.10–0.75 μg/kg LOQs: 0.33–2.50 μg/kg	[72]
Tea	16 PAHs	UAE with *n*-hexane, purification by dispersive SPE using PSA and C18 as sorbents	HPLC-UV-FLD	Waters PAH C18 column (25 cm × 4.6 mm i.d., 5 μm) at 27 °C with a gradient mobile phase of acetonitrile and water and UV detection wavelength of 230 nm	Recoveries: 71.5–118% RSDs ˂ 10% LOQs: 0.4–3.0 μg/kg	[73]
Dried fish	16 PAHs	SOX with methylene chloride, purification by GPC with SX-3 Bio Beads as a sorbent and a chromatography column with Al_2_O_3_ and silica gel as sorbents	GC-MS/MS	TG-5MS capillary column (30 m × 0.25 mm i.d., 0.25 μm); programmed temperature; splitless injection; inlet and ion source temperature at 270 °C and 280 °C, respectively	Recovery: 50–126%	[104]
Aquatic products (grass carp, *Macrobrachium rosenbergii*, *Eriocheir sinensis*)	16 PAHs	SOX with cyclohexane/methylene chloride (1/1, *v*/*v*), followed by GPC cleanup	GC-MS	DB-35MS capillary column (30 m × 0.25 mm i.d., 0.25 μm); programmed temperature; splitless injection; both inlet and ion source temperature at 260 °C; transfer line and quadrupole temperature at 280 °C and 150 °C, respectively	Recoveries: 70.3–126.4% RSDs: 0.14–8.91% LODs: 0.017–0.171 μg/kg LOQs: 0.051–0.489 μg/kg	[105]
Aquatic products (marine fish, Shrimp, Sea crab)	16 PAHs	UAE with methylene chloride, purification by an SPE cartridge using C18 and PSA as sorbents	GC-MS	HP-5MS capillary column (30 m × 0.25 mm i.d., 0.25 μm); programmed temperature; splitless injection; inlet, transfer line, and ion source temperature at 250 °C, 280 °C, and 230 °C, respectively	Recoveries: 77.5–107.4% RSDs: 1.8–8.4% LODs: 0.12–0.25 μg/kg LOQs: 0.4–0.82 μg/kg	[109]
Herbal tea	10 PAHs	QuEChERS (acetonitrile, anhydrous MgSO_4_ and NaCl, PSA and strong anion exchange sorbents) and LLE with hexane	GC-FID	PAH capillary column (30 m × 0.25 mm i.d., 0.36 mm); programmed temperature; split mode at split ratio of 10:1; inlet temperature at 270 °C	Recoveries: 77–85% LODs: 0.08–0.17 μg/kg LOQs: 0.24–0.51 μg/kg	[121]
Charcoal-grilled chicken drumsticks	16 PAHs	QuEChERS (acetone, anhydrous MgSO_4_ and NaCl, PSA and endcapped octadecylsilane silica gel particle sorbents)	HPLC-FLD	Pinnacle II PAH column (15 cm × 3 mm i.d., 4 μm) at 35 °C with a gradient mobile phase of water and acetonitrile containing 4% tetrahydrofuran and UV detection wavelength of 254 nm	Recoveries: 67–114% RSDs: 1–21% LODs: 0.004–0.25 μg/kg LOQs: 0.01–0.75 μg/kg	[124]
Kebabs	16 PAHs	UAE with acetonitrile and acetonitrile–saturated hexane, cleanup by a PAH-MIP column	HPLC-FLD-DAD	SB C18 column (25 cm × 4.6 mm i.d., 5 μm) at 30 °C with a gradient mobile phase of acetonitrile and water	Recoveries: 81–104% RSDs: 0.95–5.84% LODs: 0.33–3.30 μg/kg LOQs: 1.0–10.0 μg/kg	[145]
Oil-tea camellia seed oil	16 PAHs	Vortex extraction with hexane, cleanup by a PAH-MIP column	GC-MS/MS	HP-5MS capillary column (30 m × 0.25 mm i.d., 0.25 μm); programmed temperature; splitless injection; both inlet and transfer line temperature at 280 °C; ion source and quadrupole temperature at 230 °C and 150 °C, respectively	Recoveries: 71.5–116.3% RSDs: 1.5–13.8% LODs: 0.01–0.20 μg/kg LOQs: 0.04–0.65 μg/kg	[146]
Tea infusion	BaA, BbF, and BaP	Sample solution mixed with gelatin aerogel tablet were vortexed and then desorbed by hexane	HPLC-DAD	UPS C18 column (15 cm × 4.6 mm i.d., 5 μm) at 25 °C with an isocratic mobile phase of acetonitrile/water (95/5, *v*/*v*) and DAD detection wavelength of 256 nm	Recoveries: 70.1–119.3% RSDs ≤ 6.3% LODs:0.0017–0.0021 μg/L	[151]
Fried food	BaP, BbF, and BaA	Saponification and SPE using β-cyclodextrin functionalized graphene oxide-grafted silica as sorbent	HPLC-DAD	C18 column (15 cm × 4.6 mm i.d., 5 μm) at 30 °C with an isocratic mobile phase of methanol/water (80/20, *v*/*v*) and DAD detection wavelength of 254 nm	Recoveries: 91.2–109.1% RSDs: 1.6–11.8% LODs: 0.1–0.3 μg/L	[152]
Coffee (ground coffee, infusion, and coffee grounds)	Nap, Ant, Aceny, BghiP, BaP, Chr, Fluo, Flu, and Pyr	DI-SPME using a polyacrylate-coated fused silica fiber (85 μm)	GC-MS	SPB-5 fused silica capillary column (30 m × 0.25 μm i.d., 0.25 μm); programmed temperature; splitless injection; both inlet and ion source temperature at 250 °C; transfer line temperature at 300 °C	Recoveries: 59–99% RSDs: 3–11% LODs: 0.3–16.6 μg/kg LOQs: 1.0–55.3 μg/kg	[159]
Coffee and tea	16 EPA PAHs	HS and DI-SPME using a fiber by coating clay–chitosan and dicationic ionic liquid onto a stainless steel wire	GC-MS	TG-5MS capillary column (30 m × 0.25 mm i.d., 0.25 μm); programmed temperature; splitless injection; inlet, ion source, and transfer line temperature at 250 °C, 280 °C, and 250 °C, respectively	Recoveries: 87.5–112% RSDs: 2.5–6.5% LODs: 0.1–10 ng/L	[161]
Honey	Acen, Phen, Flu, Ant, and Pyr	HS-SPME using covalent organic framework/Ti_3_C_2_T_x_ composites as coatings	GC-FID	HP-5 capillary fused silica column (30 m × 0.32 mm i.d., 0.25 μm) and programmed temperature	Recoveries: 73.2–112% RSDs ≤ 9.4% LODs: 0.2–0.6 μg/kg LOQs: 0.6–2.0 μg/kg	[162]
Grilled meat	16 PAHs and 36 PCBs	QuEChERS (ethyl acetate/acetone/isooctane (2/2/1, *v*/*v*/*v*), NaCl and ammonium formate, MgSO_4_, and CH_3_COONa, PSA and Z-Sep+ made up of C18 and silica coated with zirconium dioxide at a ratio of 2:5 as sorbents)	GC-MS	HP-5MS capillary column (30 m × 0.25 mm i.d., 0.25 μm); programmed temperature; splitless injection; ion source and transfer line temperature at 230 °C and 280 °C, respectively	Recoveries: 72.5–119.5% RSDs: 1.3–16.8% LOQs: 0.5–2 μg/kg	[168]
Coffee, tea and water	15 PAHs	μ-QuEChERS (acetonitrile, anhydrous MgSO_4_ and NaCl, PSA, and C18 sorbents) followed by enrichment using air-assisted DLLME with diethyl carbonate as the extraction solvent	GC-MS	DB-5MS capillary column (30 m × 0.25 mm i.d., 0.25 μm); programmed temperature; splitless injection; ion source temperature at 230 °C	Recoveries: 90–103% RSDs: 0.2–5.9% LODs: 0.01–2.10 μg/kg LOQs: 0.03–6.36 μg/kg	[169]
Bread	10 PAHs	QuEChERS (acetonitrile, anhydrous MgSO_4_ and NaCl, PSA sorbent)	GC-MS	DB-5MS capillary column (30 m × 0.25 mm i.d., 0.25 μm); programmed temperature; splitless injection; inlet, quadrupole mass analyzer, transfer line, and ion source temperature at 300 °C, 100 °C, 280 °C, and 230 °C, respectively	Recoveries: 92–106% RSDs: 3–7% LODs: 0.14–0.78 μg/kg LOQs: 0.46–2.60 μg/kg	[170]
Pork meat	16 PAHs	QuEChERS with acetonitrile and Agilent 5982–6650 Extract Pouches, purification by dispersive SPE using PSA as sorbent	GC-MS	SLB-5MS capillary column (30 m × 0.32 mm i.d., 0.25 μm); programmed temperature; splitless injection; both inlet and transfer line temperature at 300 °C; ion source temperature at 220 °C	Recoveries: 71–120% RSDs: 1.7–7.6% LODs: 0.27–5.60 μg/kg LOQs: 0.81–16.8 μg/kg	[171]
Bread and potato Tahdig	16 PAHs	Sample mixed with methanol, refluxed with potassium hydroxide and distilled water, then *n*-hexane is added, and purified by a Sep-Pak cartridge with methanol/*n*-hexane (1/1, *v*/*v*) as the elution solvent	GC-MS	DB-5MS capillary column (30 m × 0.25 mm i.d., 0.25 μm); programmed temperature; pulsed splitless mode; inlet, transfer line, ion source, and quadrupole temperature at 290, 300, 230, and 150 °C, respectively	Recoveries: 86.2–100.5%	[172]
Tea	16 PAHs	UAE with hexane, purification by dispersive SPE using PSA and C18 as sorbents	GC-MS	DB-5MS capillary column (30 m × 0.25 mm i.d., 0.25 μm); programmed temperature; 20% split injection mode; ion source and transfer line temperature at 230 °C and 280 °C, respectively	Recoveries: 83.2–108.7% RSDs: 0.19–6.9% LODs: 0.10–0.28 μg/kg LOQs: 0.35–1.01 μg/kg	[173]
Honey	33 PAHs	Sample dissolved in 10% methanol/deionized water and extracted using an SPE cartridge and eluting with ethyl acetate	GC-MS/MS	Zebron™ column (20 m × 0.18 mm i.d., 0.18 μm); programmed temperature; pulsed splitless mode; injector port and interface temperature at 290 °C and 250 °C, respectively	Recoveries: 41–115% RSDs: 17–40% LOQs: 0.5 μg/kg	[175]
Seafood	24 PAHs and 18 halogenated PAHs	UAE with acetonitrile/acetone (3/2, *v*/*v*), purification by an EMR lipid tube	GC-MS/MS	DB-EUPAH column (20 m × 0.18 mm i.d., 0.14 μm); programmed temperature; splitless injection; inlet, transfer line, ion source, and quadrupole at 280 °C, 300 °C, 230 °C, and 150 °C, respectively	Recoveries: 70.5–116.9% RSDs ≤ 13.1% LODs: 0.01–0.2 μg/kg LOQs: 0.03–0.67 μg/kg	[176]
Barbecued meat and meat substitutes	6 PAHs and 8 oxygenated PAHs	PLE with acetonitrile/ethyl acetate (1/3, *v*/*v*), purification by an SPE cartridge using Florisil, zirconia/silica, and C18 as sorbents	GC-HRMS	PAH C18 capillary column (60 m × 0.25 mm i.d., 0.10 μm); programmed temperature; splitless injection; inlet, transfer line, and ion source temperature at 280 °C, 270 °C, and 260 °C, respectively	Recoveries: 72–109% LODs: 0.03–0.17 μg/kg LOQs: 0.10–0.55 μg/kg	[177]
Roasted pork meat	BaA, Chr, BaP, BbF, BkF, DiBahA, and BghiP	Homogenization and alkaline hydrolysis of samples with 1 mol/L KOH solution, purification by an SPE cartridge using propyl sulfonic as sorbent	HPLC-FLD	Hypersil Green PAH column (25 cm × 4.6 mm i.d., 5 μm) at 40 °C with an isocratic mobile phase of acetonitrile/water (84/16, *v*/*v*)	Recoveries: 61–96% RSDs: 7.8–15.9% LODs: 0.003–0.006 μg/kg LOQs: 0.01–0.02 μg/kg	[196]
Tea infusion	BaP, BaA, BbF, and Chr	QuEChERS (acetonitrile containing 1% acetic acid, anhydrous MgSO_4_ and NaCl, PSA, endcapped octadecyl siloxane, and carbon sorbents)	HPLC-FLD	Pinnacle II PAH column (15 cm × 3.0 mm i.d., 4 μm) with a gradient mobile phase of water and acetonitrile containing 4% tetrahydrofuran	LODs: 0.02–0.11 μg/kg LOQs: 0.08–0.38 μg/kg	[197]
Tea and tea infusion	BaA, Chr, BbF, and BaP	Vortex extraction with ethyl acetate, purification by dispersive SPE using silica gel and PSA as sorbents	HPLC-FLD	Vydac 201 TP54 C18 column (25 cm × 4.6mm i.d., 5 μm) at 30 °C with a gradient mobile phase of acetonitrile and water	Recoveries: 54–99% RSDs: 1–21% LODs: 0.03–0.3 μg/kg LOQs: 0.1–0.5 μg/kg	[198]
Milk	BaP, BaA, and BbF	Saponification and DLLME using chloroform as the extracting solvent and acetonitrile as the disperser solvent	HPLC-UV	PerfectSil ODS-3 HD column (12.5 cm × 4 mm i.d., 5 μm) at 25 °C with an isocratic mobile phase of acetonitrile/water (90/10, *v*/*v*) and UV detection wavelength at 254 nm	Recoveries: 88.4–95.2% RSDs: 3.7–9.7% LODs: 0.06–0.18 ng/mL LOQs: 0.18–0.56 ng/mL	[199]
Cooked chicken and roasted coffee	16 PAHs	QuEChERS (acetonitrile containing 1% acetic acid, MgSO_4_, CH_3_COONa, and PSA sorbent)	HPLC-FLD	Spherisorb ODS2 column (25 cm × 4.6 mm i.d., 5 μm) at 30 °C with an isocratic mobile phase of acetonitrile and water (90: 10, *v*/*v*)	Recoveries: 52.6–103.9% RSDs < 22% LODs: 0.03–0.6 ng/mL LOQs: 0.09–1.8 ng/mL	[200]
Grilled meat	15 PAHs	Extraction with diatomaceous earth columns and propylsulfonic acid columns using dichloromethane as the elution solvent, and cleanup by a column chromatography containing silica gel and elution with *n*-hexane/dichloromethane (60/40, *v*/*v*)	HPLC-UV-FLD	PAH column (25 cm × 4.6 mm i.d., 5 μm) with a gradient mobile phase of water and acetonitrile and UV detection wavelength at 254 nm	Recoveries: 13.7–132.6% LODs: 0.025–5 μg/kg LOQs: 0.075–15 μg/kg	[201]

## 6. Conclusions

We have reviewed here the research related to PAHs’ impact on crops and their occurrence, sources, and methods of detection in food. The existing studies indicated that PAHs present a dual impact on crop growth. They can negatively affect crop development; however, they enhance the quality and yield of agricultural produce at specific concentrations. The PAHs that occur in foods are primarily derived from the uptake and accumulation in crop leaves, as well as from food processing methods such as frying, barbecuing, smoking, etc. The prevalence of PAHs in food has become a widespread concern, due to their frequent identification in food products from numerous countries. This is particularly true for smoked foods, which require heightened attention due to the elevated levels of PAHs often found in them. The toxicity of PAHs to humans and agricultural crops has made PAHs monitoring a hot topic. We evaluated here the sample preparation techniques, which is a pivotal step in the extraction and cleanup of food matrices. Each technique offers specific application scenarios, scopes, and technical merits. Traditional methods (e.g., SOE and LLE) have shown inefficiency and high solvent consumption, which contravenes the tenets of green chemistry. Consequently, they are increasingly being supplanted by innovative technologies such as SPME and QuEChERS, which promise superior efficiency and environmental compatibility. The adoption of these new technologies is on the rise. Regarding detection, GC-MS and HPLC represent the prevailing analytical techniques; however, GC-MS may encounter difficulties in separating specific PAH isomers, while HPLC may have suboptimal qualitative capabilities. The use of ELISA and SERS techniques has augmented the sensitivity, speed, and convenience of PAH detection. Nonetheless, these techniques are yet to be universally standardized, and they currently only facilitate the detection of individual PAHs or a limited number thereof. Moreover, samples are necessitated to undergo a laborious preprocessing phase prior to detection. In addition, the employment of these two methods in food is still lacking.

In light of the current research, it is recommended that further studies be undertaken to explore the impact of PAH stress on a broader range of crops to establish tolerance thresholds and to elucidate the underlying mechanisms by which PAHs affect various crop plants. This will provide valuable insights into the cultivation and regulation of crops. Furthermore, there should be a focus on continually monitoring PAH levels in food and establishing a fundamental database. Further, removal techniques for PAHs should be developed to minimize contamination in food. Additionally, emphasis must be placed on developing green, efficient, and intelligent technologies to simplify or eliminate the sample preparation for analyzing multiple PAHs simultaneously. Concurrently, efforts must be intensified to develop portable and rapid detection devices for on-site and real-time monitoring of PAHs in various foods. It is hoped that this review can provide reference for future research endeavors.

## Figures and Tables

**Table 1:** Physical–chemical properties of the 16 EPA PAHs.

PAHs	Abbreviation	Formula	Aromatic Ring	Water Solubility (mg/L)	Melting Point (°C)	Boiling Point (°C)	Vapor Pressure (mm Hg)
Naphthalene	Nap	C_10_H_8_	2	31	80.26	218	0.087
Acenapthene	Acen	C_12_H_10_	3	3.8	95	96	4.47 × 10^−3^
Acenaphthylene	Aceny	C_14_H_10_	3	16.1	92–93	265–275	0.029
Anthracene	Ant	C_14_H_10_	3	0.045	218	340–342	1.75 × 10^−6^
Phenanthrene	Phen	C_14_H_10_	3	1.1	100	340	6.8 × 10^−4^
Fluorene	Flu	C_13_H_10_	3	1.9	116–117	295	3.2 × 10^−4^
Fluoranthene	Fluo	C_16_H_10_	4	0.26	110.8	375	5.0 × 10^−6^
Benzo[a]anthracene	BaA	C_20_H_12_	4	0.011	158	438	2.5 × 10^−6^
Chrysene	Chr	C_18_H_12_	4	1.5 × 10^−3^	254	448	6.4 × 10^−9^
Pyrene	Pyr	C_16_H_10_	4	0.132	156	393–404	2.5 × 10^−6^
Benzo[a]pyrene	BaP	C_20_H_12_	5	3.8 × 10^−3^	179–179.3	495	5.6 × 10^−9^
Benzo[b]fluoranthene	BbF	C_20_H_12_	5	8.0 × 10^−4^	215.7	480	9.59 × 10^−11^
Benzo[k]fluoranthene	BkF	C_20_H_12_	5	1.5 × 10^−3^	168.3	NS	5.0 × 10^−6^
Dibenz[a,h]anthracene	DiBahA	C_22_H_14_	6	5.0 × 10^−4^	262	NS	1.0 × 10^−10^
Benzo[g,h,i]perylene	BghiP	C_22_H_12_	6	2.6 × 10^−4^	273	550	1.03 × 10^−10^
Indeno[1,2,3-c,d]pyrene	IP	C_22_H_12_	6	0.062	163.6	530	10^−16^–10^−10^

NS, not specified.

**Table 3:** The levels of PAHs in food and products of some countries.

Nation	Type of Food	PAHs	Concentration Range (μg/kg)	Ref.
China	Youtiao	16 EPA PAHs	9.90–89.97	[71]
	Tea	22 PAHs	136.99–462.51	[72]
	Tea	16 EPA PAHs	11.4–1251	[73]
	Wild marine fishes	16 EPA PAHs	34.7–108	[74]
	Smoked meat	16 EPA PAHs	14.4–56.3	[75]
Nigeria	Oils and tomato sauces from canned fish	16 EPA PAHs	101–698	[76]
	Tea	16 EPA PAHs	0.76–44.57	[77]
	Smoked fishes	16 EPA PAHs	694–3585	[78]
	Vegetables (e.g., white spinach and lettuce)	16 EPA PAHs	100–5000	[79]
Brazil	Honey	16 EPA PAHs	1.4–23.3	[80]
	Chocolate	PAH4	8.38–41.58	[81]
Iran	Edible vegetable oils	13 PAHs	12.63–182.80	[66]
	Fish and prawn	16 EPA PAHs	2.3–13.81	[82]
	Coffee	16 EPA PAHs	13.00–20.78	[83]
Japan	Grilled foods	12 PAHs	0.062–1102	[57]
	Smoke-dried bonito	29 PAHs	419–1070	[84]
Latvia	Cereal products	PAH4	0.22–1.62	[85]
Tanzania	Smoked and sun-dried fishes	13 PAHs	80–33,900	[86]
Ghana	Smoke-cured fish	16 EPA PAHs	510.59–1461.79	[87]
Italy	Seafood	16 EPA PAHs	20.26–282.2	[88]
Bangladesh	Milk	16 EPA PAHs	0.5497–1.077	[89]
Pakistan	Vegetables	16 EPA PAHs	51.6–402	[90]
Saudi Arabia	Vegetables and fruits	16 EPA PAHs	10.11–798.21	[91]
Argentina	Tea	16 EPA PAHs	509.7–2746.5	[92]
Turkey	Tea	15 PAHs	212.2–953.9	[93]
	Honey	14 PAHs	464.32–650.24	[94]
India	Bread, biscuits, tea, coffee, oils, chocolates, grapes, pepper, and fishes	16 EPA PAHs	0.18–61,967	[95]
Egypt	Grilled, pan-fried, and boiled meat	15 PAHs	12.21–72.16	[96]
Romania	Smoked fish and smoked cheese	16 EPA PAHs	8.54–56.3	[97]
Poland	Tea	16 EPA PAHs	41.5–2910.2	[98]
	Tea infusion		52.9–2226.0 ng/L	
Canada	Mussel, clam, and cockle	17 PAHs	510–17,550	[99]
Korea	Processed foods and their raw materials (e.g., cereals, nuts, fruit, meat, fish, beverages, and seasonings)	8 PAHs	0.08–11.97	[100]
Togo	Fish	12 PAHs	5.24–149.0	[101]
Ghana	Fish	28 PAHs	71–481	[102]
	Vegetables (e.g., Chinese cabbage, lettuce, and garden egg leaves)	16 EPA PAHs	3.16–9.29	[103]

## Data Availability

Not applicable.
